# The Role of Magnetic Resonance Imaging and Cardiac Computed Tomography in the Assessment of Left Atrial Anatomy, Size, and Function

**DOI:** 10.1155/2015/247865

**Published:** 2015-07-07

**Authors:** Petr Kuchynka, Jana Podzimkova, Martin Masek, Lukas Lambert, Vladimir Cerny, Barbara Danek, Tomas Palecek

**Affiliations:** ^1^Second Department of Medicine-Department of Cardiovascular Medicine, First Faculty of Medicine, Charles University in Prague and General University Hospital in Prague, Prague, Czech Republic; ^2^International Clinical Research Center, St. Anne's University Hospital in Brno, Brno, Czech Republic; ^3^Department of Radiology, First Faculty of Medicine, Charles University in Prague and General University Hospital in Prague, Prague, Czech Republic

## Abstract

In the last decade, there has been increasing evidence that comprehensive evaluation of the left atrium is of utmost importance. Numerous studies have clearly demonstrated the prognostic value of left atrial volume for long-term outcome. Furthermore, advances in catheter ablation procedures used for the treatment of drug-refractory atrial fibrillation require the need for detailed knowledge of left atrial and pulmonary venous morphology as well of atrial wall characteristics. This review article discusses the role of cardiac magnetic resonance and computed tomography in assessment of left atrial size, its normal and abnormal morphology, and function. Special interest is paid to the utility of these rapidly involving noninvasive imaging methods before and after atrial fibrillation ablation.

## 1. Introduction

In recent years there has been increased interest in the comprehensive evaluation of the left atrium (LA). The main role of the LA is to modulate left ventricular (LV) filling through several mechanical functions: reservoir function, conduit function, and active contraction [1]. Preserved active LA pump function contributes up to 30% of the total LV stroke volume; its loss due to atrial fibrillation or ventricular pacing may lead to symptomatic deterioration, especially in the setting of LV dysfunction [2]. Left atrial size increases for a number of reasons: due to pressure overload caused by LV filling pressures or mitral stenosis; due to volume overload associated with mitral regurgitation or high cardiac output; or due to atrial fibrillation (AF). Dilatation of the LA has been repeatedly shown to be a strong marker of adverse cardiovascular outcomes [3–6]. The introduction of catheter ablation techniques for the treatment of drug-resistant AF has represented an important stimulus for advances in the field of multimodality imaging of the LA.

The aim of this paper is to give a detailed overview on the utility of cardiac magnetic resonance (CMR) and cardiac computed tomography (CT) in the assessment of LA anatomy, size, and function.

## 2. Cardiac Magnetic Resonance 

### 2.1. Basic Aspects of Cardiac Magnetic Resonance Imaging

**Figure 1:**
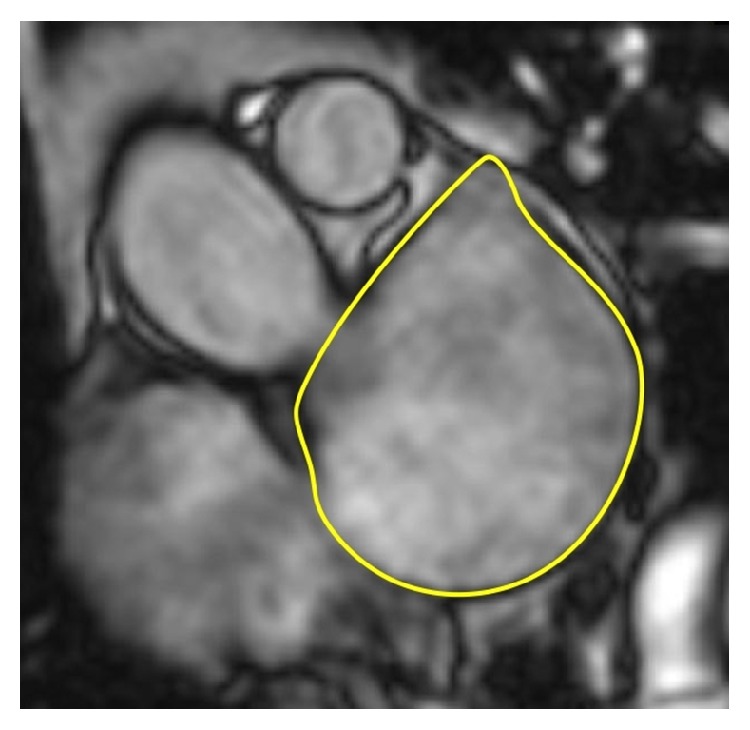
Representative slice demonstrating the tracing of the left atrial boundary at ventricular end-systole in short-axis view on steady-state free precession magnetic resonance image. The left atrial volume is then calculated using the disk area summation method (Simpson's method) from contiguous slices covering the whole left atrial cavity.

**Figure 2:**
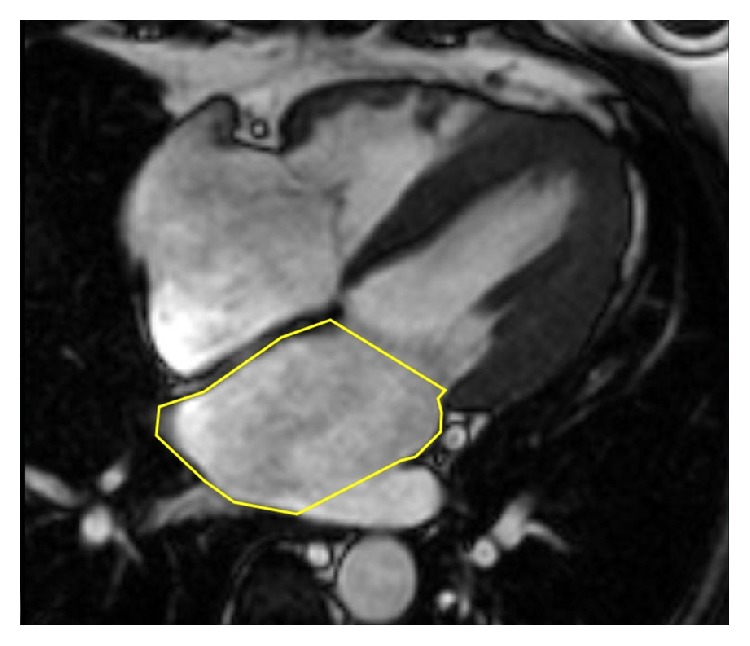
Measurement of the left atrial area at ventricular-end systole in four-chamber view on steady-state free precession magnetic resonance image.

**Figure 3:**
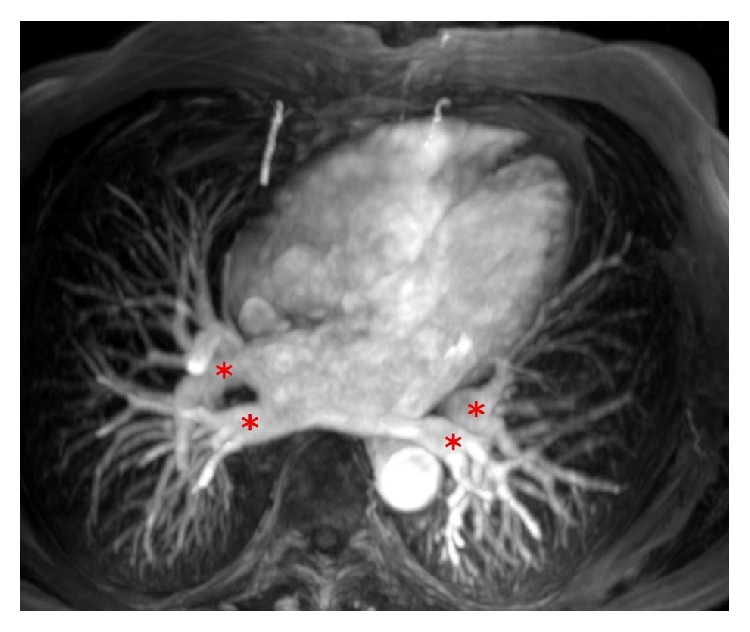
Normal anatomy of four pulmonary veins (red stars) on three-dimensional contrast-enhanced magnetic resonance angiography.

Cardiac magnetic resonance has been considered the gold standard for assessment of the heart chambers [7]. Its high spatial and temporal resolution allows accurate assessment of the morphology and function of both the atria and ventricles in healthy subjects as well as in patients with various structural heart diseases. The advantage of CMR is the absence of radiation exposure and the ability to characterize tissue composition. Cine CMR images are obtained as end-expiratory breath-hold and ECG-triggered acquisitions with steady-state free precession sequences. Consecutive multislice acquisitions of the entire LA in short-axis view with either manual tracing of the atrial wall or automated border detection form basis for the assessment of LA volumes based on Simpson's method of discs (Figure 1). Slice thickness is usually between 2.5 and 5 mm, with a temporal resolution of 25 to 50 ms. Alternatively, LA volumes may be measured using the biplane area-length method in the horizontal and vertical long axes [8]. Left atrial area shall be measured in four-chamber view (Figure 2). The uniqueness of CMR as compared to echocardiography as well as cardiac CT is its ability of tissue characterization and scar imaging using late gadolinium enhancement (LGE) technique [8]. Inversion recovery gradient echo sequences in long and short axes are acquired 10–20 minutes after the injection of gadolinium-based contrast agent. Gadolinium-enhanced three-dimensional magnetic resonance angiography provides detailed information on pulmonary vein anatomy in relation to the LA cavity (Figure 3). If contrast agent administration is contraindicated, three-dimensional steady-state free precession angiography may be a viable option for how to assess anatomy of pulmonary veins and the LA.

The main limitations of CMR examination are the length of the complete heart imaging procedure (about 45 minutes), the financial burden, possible evocation of claustrophobia, limited availability, and the incompatibility of certain prosthetic materials [9]. Furthermore, gadolinium-containing contrast agents are contraindicated in subjects with glomerular filtration rate < 30 mL/min/1.73 m^2^ due to the risk of developing nephrogenic systemic sclerosis.

### 2.2. Left Atrial Morphology

Accurate visualization of LA morphology is essential in many clinical situations, such as assessment of the feasibility of radiofrequency ablation (RFA) in patients with atrial fibrillation (AF), planning cardiovascular surgery, evaluation of left atrial appendage (LAA) thrombosis, and evaluation of various other pathological masses like tumours and infiltration of the cardiac wall. Conventionally, transthoracic echocardiography (TTE) has been the most useful and practical method for atrial investigation in daily clinical practice due to its wide availability and cost-benefit ratio. Cardiac CT currently represents a gold standard for assessment of the anatomy of the LA complex, LAA shapes and lobes, LA wall thickness, atrial septal anomalies such as septal defects, patent foramen ovale, and diverticula. Cardiac CT is generally performed rapidly, with acceptable temporal resolution and high spatial resolution. Nevertheless, standard CMR techniques also allow assessment of some LA morphological and functional parameters with reasonable accuracy which may be of particular importance in patients undergoing RFA of atrial fibrillation as described in more detail below.

### 2.3. Left Atrial Masses

Cardiac magnetic resonance allows excellent characterization of various intra-atrial masses. The technique enables superior tissue characterisation and thus differentiation between cardiac tumours and thrombi. Cardiac magnetic resonance can also be helpful in differentiating between benign and malignant tumours. In a study comprising 116 patients with various cardiac masses, Pazos-López et al. used CMR to show that tumours tend to be larger, heterogeneous, and more mobile compared to thrombi [10]. The presence of LGE and hyperintensity on T2-weighted images is also typical for tumours rather than for thrombi. However, the accuracy of CMR in distinguishing between benign and malignant tumors was moderate in this study. Malignant tumours were larger and exhibited contrast first-pass perfusion and the presence of LGE more frequently. Myxomas represent the most common benign tumours in LA, followed by rhabdomyomas and fibromas. Myxomas are typically mobile, oval masses attached to the atrial septum, and they tend to have heterogeneous CMR signal with a typical LGE pattern [10, 11]. Less frequent LA mass findings include malignant tumours such as lymphomas and sarcomas, and nonneoplastic lesions like thrombi, infectious vegetations, and pericardial cysts [11].

### 2.4. Left Atrial Size

Increased LA size has been shown to be a marker of LV diastolic dysfunction as well as a predictor of conditions like myocardial infarction, heart failure, stroke, and atrial fibrillation [12–15]. In the past, LA diameters and areas were the key measured LA parameters. However, LA volume has been demonstrated to be a more robust marker of cardiovascular events [6]. Interestingly, recent literature has provided growing evidence that minimal rather than maximal LA volume should be the focus of research, as minimal LA volume better reflects LV diastolic pressure and predicts elevated pulmonary wedge pressure more accurately than maximal LA volume [16–18].

The standard evaluation of LA size in daily clinical practice is performed by transthoracic echocardiography. However, echocardiographic assessment is often inaccurate due to geometric assumptions and foreshortening of the LA cavity [18]. The accuracy of CMR volumetric measurements has been demonstrated via the tight correlation between CMR and cadaveric casts and with other imaging methods [9, 19]. Agner et al. performed an interesting head-to-head comparison of TTE, CCT, and CMR in the assessment of LA volume and function in patients with atrial fibrillation. The results of this study showed that TTE tends to underestimate the LA maximal and minimal volumes, while CT overestimates maximal and minimal LA volumes compared to CMR [20].

The current European heart failure guidelines recommend assessment of LA volume in all patients with heart failure regardless of the aetiology [21]. CMR is not a routine imaging modality for this assessment due to time and financial considerations. However, several studies have shown excellent correlation between LA CMR measurements and cardiovascular outcomes. Gulati et al. demonstrated a strong association between LA volume measured by CMR with transplant-free survival and heart failure outcomes in 483 patients with dilated cardiomyopathy [22]. Individuals with dilated cardiomyopathy with an indexed LA volume more than 72 mL/m^2^ had a threefold higher risk of death or transplantation compared to those with an indexed LA volume less than 72 mL/m^2^ [22]. In another study Jahnke et al. demonstrated that LA volumetry based on cine imaging predicted the success of pulmonary vein isolation for AF. An LA diastolic (i.e., maximal) volume of less than 112 mL was associated with sustained sinus rhythm following RFA with 80% sensitivity and 70% specificity [23].

Established CMR reference values of LA parameters are based on data derived from several studies [1, 7, 24, 25]. The normal LA volume is defined as 97 ± 27 mL with gender-specific values of 103 ± 30 mL for males and 89 ± 21 mL for females. The upper limit of LA area measured in the four-chamber view is 23 cm^2^ [26].

The MESA (Multi-Ethnic Study of Atherosclerosis) demonstrated that LA enlargement and deterioration of LA function precede the development of heart failure [27]. In this study, 112 participants with incident heart failure and 224 controls matched for sex and age were followed for 8 years. Left atrial volume and function were assessed by CMR feature-tracking. The results showed that global peak longitudinal atrial strain and higher LA minimal volume were independent markers of incident heart failure in a multiethnic population of asymptomatic individuals.

However, all these data raise an intriguing question regarding the need for highly accurate measurement of LA size [18]. For example, it is not known whether the LA appendage, which is included in the volumetric measurement by CMR but not by echocardiography, meaningfully contributes to LA volume, especially in patients with high LA volumes [18].

### 2.5. Left Atrial Function

Cardiac magnetic resonance assessment of LA function is currently not used in routine practice, but it has been attracting more research attention due to the technical development of imaging methods. The technique is highly suitable for studying parameters like LA phasic volumes and emptying fractions (total, passive, and active). Although not as high as in 2D echocardiography, the temporal resolution of steady-state free precession sequences of 25 to 50 ms is sufficient for the evaluation of LA volumes during cardiac cycle. It was demonstrated that LA passive volumetric contribution to LV filling decreases with age, while its active component increases with age. These age-related differences are augmented by dobutamine challenge, as shown by Ahtarovski et al. [28].

In a study of 210 subjects with a history of long-lasting arterial hypertension, Kaminski et al. proved a strong association between decreased LA contractile function and patient mortality, nonfatal events, and all major adverse cardiovascular events (MACE) [29]. Every 10% reduction of LA contractile function increased the risk of death, nonfatal event, or MACE by 1.5-, 1.4-, and 1.8-fold, respectively. According to multivariable analyses, the active LA contribution to LV filling was the strongest predictor of MACE.

Quite recently, Kowallick et al. reported results from a small study assessing LA deformation by CMR myocardial feature tracking. In this study 10 patients with hypertrophic cardiomyopathy, 10 individuals with heart failure with preserved LV ejection fraction, and 10 healthy volunteers were studied using CMR assessment of LA longitudinal strain and strain rate parameters derived from steady-state free precession cine images. The results showed reliable quantification of LA conduit, reservoir, and contractile function both in disease states and in healthy individuals. Moreover, excellent intra- and interobserver agreement was noted [30]. This novel approach to LA function assessment seems promising; however, larger studies on this topic need to be conducted in the future.

### 2.6. Radiofrequency Ablation of Atrial Fibrillation

Detailed assessment of the LA by CMR is increasingly used in the field of atrial arrhythmias, particularly AF. It is well known that episodes of paroxysmal AF are triggered by an ectopic beat in the muscular layer of the pulmonary veins in more than 90% of cases [31]. Further abnormal activation of the atrium is then influenced by pathological changes within the atrial wall, which shares many similarities with the pulmonary veins due to their common embryogenic development from the primitive common pulmonary vein [32].

Radiofrequency ablation of both paroxysmal and persistent AF has been performed with increasing frequency since 2003 [33]. This method is based on electrical isolation of the pulmonary veins and further ablation in LA causing linear isolations within the atrial roof and left isthmus. However, the success rate of this procedure, particularly in cases of persistent AF, is rather disappointing, with only about 57% of patients remaining in sinus rhythm after the first procedure and 71% after multiple procedures [34]. The complication rate of RFA reaches 5%, comprising mainly pulmonary vein stenosis (2–5%), tamponade (1.2%), atrioesophageal fistulas (0.05%), phrenic nerve injury, and bleeding [35]. Various imaging modalities, such as intracardiac echocardiography, cardiac CT, and CMR, can be applied to optimize the entire procedure. Cardiac CT is the most widely used method for individually tailored management of AF by sophisticated visualization of the LA in a simple and rapid manner. Nevertheless, CMR also has the potential to be used in all phases of RFA—before procedure, in real time during the procedure, and after procedure. However, its role is still experimental and research-based.

Preprocedural CMR images of the LA help to identify the anatomical relationship of the LA to other structures in thorax, thus enabling easier orientation within the LA for RFA operators. The location of the oesophagus is highly variable due to its peristaltic activity, but most often it lies directly behind the LA, very close to the left pulmonary veins, posing the risk of creating an atrial-esophageal fistula with possibly catastrophic consequences. Meng et al. showed that one-third of patients treated with RFA had oesophageal LGE on CMR performed 2 months following the ablation procedure [36]. The occurrence of LGE was not related to ablation time, type of catheter, oesophageal location, or LA size. The clinical significance of these findings is unknown; however, the LGE might represent scar tissue, with risk for delayed perforation.

The presence of thrombus in the LA appendage represents an absolute contraindication for RFA. Transoesophageal echo (TOE) is the standard method for excluding the presence of thrombus in the LA appendage with high negative predictive value (96–100%) [37]. However, TOE cannot be performed in some patients for various reasons, and CMR may represent a viable option for the exclusion of intra-atrial thrombosis. Ohyama et al. studied 50 patients with chronic nonrheumatic AF and a history of stroke who underwent both TOE and CMR. In the study, thrombosis was detected by CMR in three patients who were declared thrombus-free by TOE [38]. This finding might be either due to the higher sensitivity of CMR compared to TOE or due to its lower specificity caused by respiratory motion artefacts during CMR. Furthermore, a significant correlation has been demonstrated between CMR and TOE measurements of peak emptying velocity of the LA atrial appendage, which is a predictor of thrombus formation [37].

Preprocedural identification of pulmonary vein anomalies—their number, location, or branching–could potentially decrease the recurrence rate of AF and the risk of pulmonary vein stenosis. Pulmonary vein stenosis occurs in about 2–5% of patients undergoing RFA [39]. It is caused by intimal proliferation and myocardial necrosis induced by radiofrequency energy. Stenotic lesions are more likely to develop in smaller veins after extensive ablation performed deeper in the pulmonary vein trunk [40, 41]. Pulmonary vein stenosis may be completely asymptomatic or it can lead to pulmonary hypertension and hypoperfusion of the affected lung segments, manifesting with cough and dyspnoea [39]. Pulmonary vein angioplasty is usually sufficient to relieve patients' symptoms and it is required in about 50% of cases [42]. Although CMR as well as CT is able to visualize pulmonary vein stenosis with high accuracy, TOE is usually the first method performed when suspicion for this postprocedural complication is raised due to its wide availability and lack of radiation.

Variation in pulmonary vein anatomy occurs in approximately 40% of patients indicated to the ablation procedure [43]. Most often there is a single left common pulmonary vein or an additional right middle pulmonary vein. Right-sided pulmonary veins tend to form earlier than left-sided pulmonary veins during embryonic development and thus have more time to be incorporated into the LA, whereas left-sided pulmonary veins form later, leading to a higher frequency of a common trunk on the left side [39].

Surprisingly, several studies have failed to support the hypothesis that assessment of pulmonary vein number, location, and branching prior to RFA affects long-term efficacy of RFA [44–46]. The only parameters predictive of AF recurrence seem to be the diameter of the pulmonary ostia, particularly their cross-sectional area, and increased pulmonary vein contraction [47, 48]. The measurement of the pulmonary vein ostia is complicated, as there is no clear anatomical border between the pulmonary veins and the LA. The ostia are ovoid and their size varies during the cardiac cycle. Maximal diameter, perimeter, and cross-sectional area can be measured by CMR in the sagittal plane. In an important study conducted by Syed et al., measurements of pulmonary vein ostia were performed by CT, intracardiac echocardiography, TOE, and venography, resulting in different numbers and positions of pulmonary veins, with poor correlation of diameter measurements among the imaging modalities [49]. Patients with the highest cross-sectional areas measured by CMR tend to have recurrent AF after ablation independently of the type of AF and LA size. Furthermore, it seems that preservation of pulmonary vein contraction on follow-up CMR might be an independent predictor of incomplete ablation [48].

**Figure 4:**
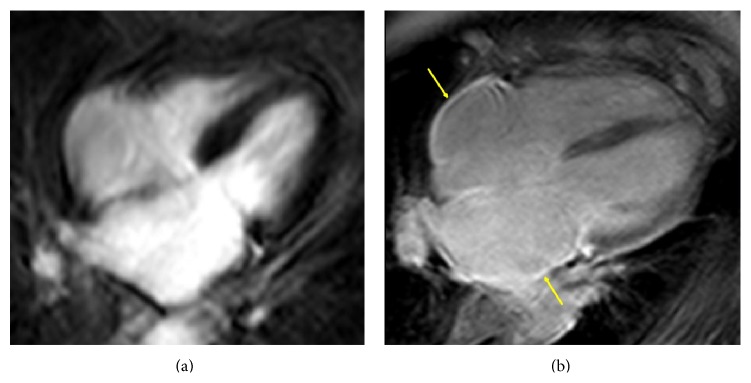
The examples of inversion recovery gradient echo sequencies in four-chamber view demonstrating the absence (left) and the presence of late gadolinium enhancement (right; arrows) in atrial walls.

Extensive structural remodelling of the LA seems to play an important role in the prediction of the phenotype of AF, recurrence of arrhythmia, thromboembolic events, and mortality [9]. Left atrial remodelling can be predicted using CMR by the severity of atrial enlargement and by the extent of LGE [9, 50]. Left atrial LGE results from altered washout kinetics of gadolinium from fibrotic regions relative to normal surrounding tissue (Figure 4) [51, 52].

A unique scoring system called The Utah Staging System has been developed to quantify the degree of LA structural remodelling based on the extent of LGE: Utah stage I is defined by less than 5% LGE, Utah stage II as 5–20% LGE, Utah stage III as 21–35% LGE, and Utah stage IV as more than 35% LGE [53]. A significant correlation between the extent of LGE and clinical outcome has been repeatedly demonstrated. In a study by Daccarett et al. the recurrence rate of AF within 8 months after RFA was 0% in patients with Utah stage I and 56% in Utah stage IV [50]. Another study by Oakes et al. documented AF recurrence after RFA in 14% of patients with minimal LGE of the LA wall, in 43.3% of patients with moderate LGE, and in 75% of patients with extensive LGE of the LA wall [53]. A high scar burden preablation thus predicts a high recurrence rate of AF, as well as increased difficulty and length of the ablation procedure. Oakes et al. also noticed a relationship between the location of scarring and the extent of LGE. Patients with mild to moderate LGE of the LA were particularly affected on the posterior LA wall and interatrial septum, while patients with extensive enhancement had scarring detectable in all parts of the LA wall. The same authors also showed a statistically significant correlation between LA volume and the extent of LGE [53].

Scarring of the LA wall visualized by LGE also seems to be associated with increased risk of thromboembolism. According to Wylie et al., this risk is related to the decreased contractile function of the LA, even in the absence of AF [54]. Daccarett et al. showed that patients with mild remodelling of the LA, defined as an extent of LGE less than 8.5%, experienced low rates of thromboembolic complications (LGE 2.8%). On the other hand, more than 50% of patients with severe enhancement (LGE >21.1%) experienced an ischemic event. Furthermore, a higher extent of scarring was noted in patients with a prior history of stroke compared to patients with no history of stroke [50].

Based on these findings, CMR could aid in the stratification of AF patients, allowing for optimal choice of treatment and individualized care. Patients with low grade LGE are likely to benefit from the standard ablation protocol, while patients with extensive LA fibrosis should probably be treated conservatively with adequate rate-control management with long-term anticoagulation or with an extensive ablation strategy (i.e., ablation of the posterior wall and septal LA debulking) [50, 53]. Nevertheless, it must be clearly stated that the evaluation of the presence and the extent of LGE in the LA wall is very difficult due to its thinness and requires a high expertise in CMR.

A further challenge is the use of real-time CMR for online navigation of the ablation catheter. The use of CMR could give the operator immediate feedback of lesion formation during ablation, thus distinguishing between true scar formation and tissue oedema that leads to a temporary electrical gap, with delayed pulmonary vein reconnection [37]. In addition, the use of CMR could reduce possible complications by allowing real-time monitoring of the catheter position in relation to the oesophagus, pericardium, and other structures. Real-time CMR has already been tested in experimental settings and new techniques are in development. However, its widespread use is unlikely at the present time.

Postablation CMR enables visualisation of LGE as a correlate of postablation scar formation, which is likely to be predictive of RFA success [55, 56]. According to McGann et al. individuals with minimal scar formation induced by RFA had a higher rate of AF recurrence at 3-month follow-up [55]. Another important marker of the success of the ablation procedure is the creation of a circumferential pulmonary vein scar. However, this may be achieved in only about 7% of patients undergoing RFA, as shown by Badger et al. [57]. Therefore, while preablation atrial scarring predicts a lower likelihood of successful RFA, the postablation visualisation of low LGE around the pulmonary vein ostia is predictive of AF recurrence and can be helpful in planning redo procedures.

The integration of data from electroanatomic mapping with CMR or cardiac CT images into so-called hybrid maps represents an intriguing application of CMR. However, the results of existing studies seem to be quite controversial. Bertaglia et al. described significantly improved outcomes of patients after RFA using merged CMR hybrid maps [58]. On the other hand, Kistler et al. did not corroborate these data in a study using combined electroanatomic mapping with morphological information provided by cardiac CT [59]. Similarly, Caponi et al. examined the use of hybrid CMR and electroanatomic mapping and also did not observe improved clinical outcomes in neither paroxysmal nor persistent AF [60]. The number of AF recurrences was similar in both groups after 12 months of follow-up. Although the use of CMR did reduce fluoroscopy time, it had no effect on procedural success or long-term efficacy [60]. Nevertheless, reduction of X-ray exposure is an important issue because many patients require repeated procedures associated with a high radiation dose, increasing their risk of malignancy.

## 3. Cardiac Computed Tomography 

### 3.1. Basic Aspects of Cardiac Computed Tomography

Computed tomography imaging of the heart requires minimization of cardiac motion artefacts. For this reason, cardiac CT scanning is almost exclusively performed with simultaneous ECG registration. Two basic methods are recognized: prospective triggering and retrospective gating. Prospective ECG triggering is a method in which the data are acquired at a prespecified phase of the cardiac cycle (usually during the phase with minimal heart motion and therefore minimal coronary artery motion—thus in mid-diastole or in end-systole in patients with accelerated heart rate). In retrospective ECG gating, data are acquired throughout the entire cardiac cycle, and the only data obtained during the cardiac phase with the least motion artefacts are used for image reconstruction [61]. The protocol for cardiac CT is highly dependent on the technology delivered by each vendor. Some vendors attempt to decrease the radiation dose by prospective triggering and fast rotation times, while others build their protocols mainly on helical scanning and ECG pulsing, where full-dose images are acquired only during a preselected phase. Prospective ECG triggering is being performed more frequently in recent years because of its relatively low radiation dose in comparison with the retrospective gating method. However, the most important disadvantage of prospective triggering lies in the fact that images can be reconstructed only for a preselected phase of the cardiac cycle and functional assessment of the heart is thus not feasible.

In comparison with CMR, cardiac CT has worse temporal resolution (75–250 ms) but better spatial resolution (0.5–1 mm) [8].

Cardiac CT is always associated with a certain radiation dose that usually varies from 1 to 15 mSv, depending on the scanning protocol. Iodinated contrast agents are used in most cardiac CT examinations.

Beta blockers and other drugs with negative chronotropic properties, such as ivabradine and verapamil, are administered in order to achieve a target heart rate, usually less than 65 beats per minute, minimizing motion artefacts.

### 3.2. Left Atrial Morphology

The LA represents a complex structure that consists of three compartments of different embryonic origin: the anterior LA (ALA), the venous LA (VLA), and the LA appendage (LAA).

The ALA forms the anterior and central parts of the LA. The LAA is a tubular structure that bulges out superiorly and in a leftward direction. The VLA receives blood from the pulmonary veins and represents the remaining part of the LA [62].

The average wall thickness of the LA varies between subjects in sinus rhythm and those with various types of atrial fibrillation (AF). In patients with paroxysmal AF, the LA wall measured by CT is thicker compared to patients with long-standing, persistent AF as well as individuals in sinus rhythm. This is reflective of the LA wall structural remodelling that occurs in paroxysmal AF prior to LA dilatation, which occurs as the AF becomes persistent [63].

**Figure 5:**
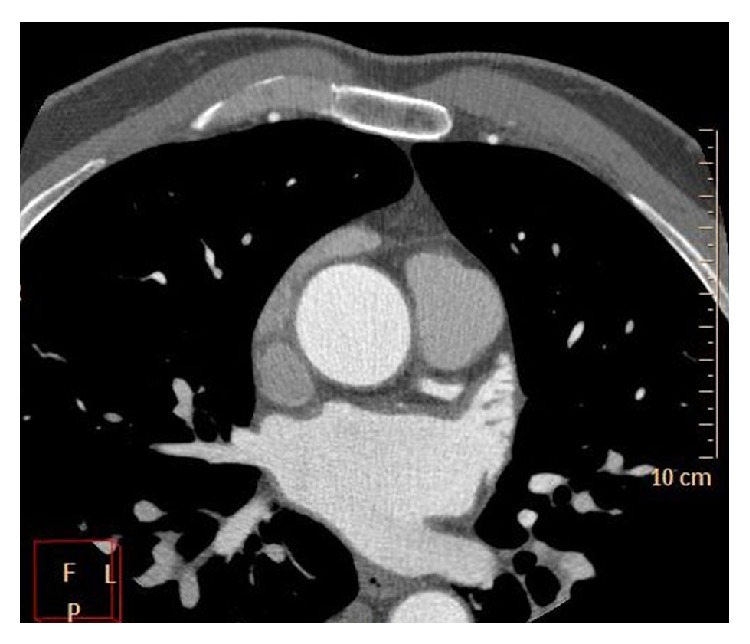
Contrast-enhanced CT image showing the most common subtype of left atrial appendage called chicken wing.

The LAA has variable shapes and number of lobes. Four different shapes have been described: cactus, chicken wing (Figure 5), windsock, and cauliflower. The cactus shape is defined as a dominant central lobe with limited overall length and one or more secondary lobes; the chicken wing shape is defined as a main lobe that bends from the proximal middle part of the LAA; the windsock shape is characterized by one dominant lobe with several secondary, or even tertiary, lobes; and the cauliflower shape describes LAA morphology with limited overall length and complex internal structures [64]. The chicken wing shape seems to be the most common morphological subtype of the LAA according to a study by Di Biase et al. [65]. In the study, 932 patients with AF were investigated either by CT or CMR, and it was demonstrated that patients with chicken wing LAA morphology were less likely to have an embolic event even after adjusting for comorbidities and CHADS_2_ score.

The risk of stroke or transient ischemic attack (TIA) due to paradoxical embolism is associated not only with atrial septal defect but also with the presence of a patent foramen ovale (PFO) which is found in approximately 25% of the general population mainly. Higher risk of stroke or TIA is also connected with interatrial septal aneurysm and with the relatively recently described atrial septal pouch. An atrial septal pouch is a lesion characterized by incomplete fusion of the septal components and thus might serve as a site for thrombus formation and subsequent embolization [66].

**Figure 6:**
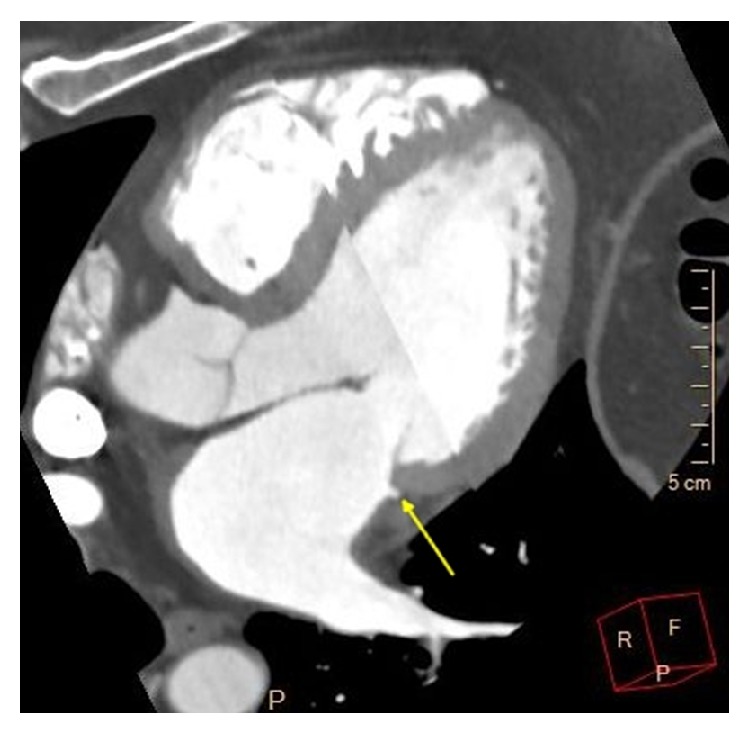
Contrast-enhanced CT image demonstrating a diverticulum (arrow) at the lateral part of the left atrium, close to the mitral valve.

Apart from abnormalities of the interatrial septum, the most common morphological variations of the LA are diverticula (Figure 6) and accessory appendages. Diverticula are defined as cyst-shaped protuberances that project outward from the heart cavity and are composed of a single muscular layer. The prevalence of diverticula ranges between 18% and 41% of subjects undergoing cardiac CT imaging, with a mean diverticular diameter of 4–6 mm [67]. The most common location of diverticula is the anterosuperior portion of the LA. The pathophysiology of their development is not clearly understood. It is hypothesized that they may be congenital, associated with incomplete development of accessory pulmonary veins or incomplete regression of cardinal veins, or they may be acquired, associated with ischemic heart disease or other heart pathologies. Diverticula are generally asymptomatic, although an association with thromboembolism and arrhythmias has been also reported [67].

Accessory LA appendages represent less common abnormalities with prevalence between 3 and 10% of subjects undergoing cardiac CT. LA accessory appendages are defined as sac-like structures with irregular contours that resemble the pectinate muscle and have narrower ostia than diverticula [68]. They are congenital and carry potential risk for thromboembolic events.

### 3.3. Left Atrial Size and Function

Left atrial size is associated with cardiovascular events and mortality [69, 70]. Cardiac magnetic resonance is considered to be the gold standard for assessment of LA size and function. Echocardiography still represents the most frequently used imaging tool for the quantification of LA size in daily clinical practice. However, with the advent of cardiac CT, information on LA morphology and size is readily available on every contrast-enhanced and even unenhanced scan of the heart. On the other hand, only CT scans acquired with retrospective gating provide information regarding LA function. Moreover, in scans obtained by prospective triggering, the LA size is assessable only in a predefined part of the cardiac cycle and so the true maximum size of the LA is usually not recorded.

In patients in sinus rhythm, there is clear evidence regarding the excellent correlation between LA size and function measured by CMR and CT. It is also well known that transthoracic echocardiography (TTE) tends to underestimate LA size and to overestimate LA systolic function in comparison with both CT and CMR [71–74].

Data regarding CT and CMR correlation in patients with ongoing AF have been sparse. Very recently Agner et al. conducted a study that included 34 patients with persistent AF, comparing TTE, CMR, and CT in the assessment of LA size expressed as LA maximal and minimal volumes (LAVmax and LAVmin, resp.) and LA systolic function represented by fractional area change (FC) [20]. Compared with CMR, CT overestimated both LA volumes (LAVmin by 8% and LAVmax by 10%). Echocardiographically derived LA volumes were underestimated in comparison with both CMR (LAVmin by 22% and LAVmax by 18%) and CT (LAVmin by 28% and LAVmax by 25%). Regarding LA systolic function, CT overestimated FC by 8%, and echocardiography overestimated FC by 23% in comparison with CMR. There was a close correlation between parameters obtained by CMR and CT. Moreover the intraobserver and interobserver agreements between these two methods were excellent. The correlation between TTE and CMR or CT was worse in all aspects of the assessment.

Recently, the advantage of unenhanced CT evaluation of the LA size was shown in a large cohort study. Mahabadi et al. reported the results of The Heinz Nixdorf Recall Study, which included 3958 subjects aged 45–75 years without coronary artery disease, stroke, atrial fibrillation, or flutter and without pacemaker or implanted defibrillator [75]. All subjects underwent non-contrast-enhanced CT scanning using the prospective triggering method. The area of the LA was measured, and major cardiovascular events during a mean follow-up of 8 years were evaluated. Endpoints were defined as coronary events, stroke, and cardiovascular death. Both LA size and its value indexed to BSA were strongly associated with the combined endpoint independently of traditional cardiovascular risk factors, even after adjusting for calcium score. The same relationship was also observed between LA size and each endpoint separately.

The LA represents a complex structure, with three compartments that are subject to differing degrees of involvement in AF patients. Park et al. conducted a study comprising 53 patients with paroxysmal AF, 43 individuals with persistent AF, and 48 control subjects in sinus rhythm who had no history of arterial hypertension, diabetes mellitus, ischemic heart disease, cardiomyopathy, or moderate to severe valvular disease. The authors assessed LA volumes indexed to body surface area (BSA) and ejection fraction of the entire LA as well as ejection fraction of each of its three components [62]. Not surprisingly, total LA volume was lowest in the control group and highest in the group of patients with persistent AF. In each of the three LA compartments, the maximum LA volume index was lowest in controls (LAA 4.8 mL/m^2^; VLA 18.3 mL/m^2^; ALA, 37.1 mL/m^2^) and highest in patients with persistent AF (LAA 9.8 mL/m^2^; VLA 30.0 mL/m^2^; ALA 67.3 mL/m^2^). The ejection fraction (EF) of the entire LA was highest in the control group and lowest in the group of patients with persistent AF (mean EF 42.89% versus 13.49%). Regarding the three LA compartments, the ejection fraction was highest in the LAA and lowest in the VLA in all three subgroups of patients.

According to this study and a report by Christiaens et al. [76], LAA parameters displayed relatively poor correlation with those of the LA, which can be explained by the fact that the LAA has a distinct pattern of contraction and a greater dependence on left ventricular function than on LA performance. The lowest regional systolic function was observed in the VLA, which may be explained by the fact that the VLA is a relatively immobile structure because the posterior LA wall is surrounded by the pericardium and parts of the great vessels of the heart. Moreover, poor contractility in the region of the pulmonary veins may contribute to the decline in VLA performance in patients with ongoing AF. It is also known that this region usually contains a high proportion of fibrosis in subjects after interventional or surgical isolation of pulmonary veins. The ALA is a mobile, smooth-walled structure, which is not fixed to the pericardium. It represents the largest compartment of the LA. Its systolic function is usually less compromised in comparison with VLA in subjects with AF and best reflects proper LA performance.

### 3.4. Left Atrial Thrombi

Thrombi are by far the most common form of LA mass and are found predominantly in the LAA. Well-known factors precipitating thrombus formation include structural and functional cardiac abnormalities, artificial cardiac implants, and hypercoagulable states [77].

Currently, TOE is considered to be the gold standard for detection of intra-atrial thrombi. The main advantages of TOE are related to its high temporal as well as spatial resolution, as well as the lack of radiation and nephrotoxic contrast agent exposure. However, TOE is a semi-invasive method, which needs to be performed with sedation in most patients and, even more importantly, is relatively highly operator-dependent.

Contrast-enhanced cardiac CT allows visualisation of the entire LA, including the LAA, and thus represents a diagnostic alternative to TOE for detection of intra-atrial thrombosis.

**Figure 7:**
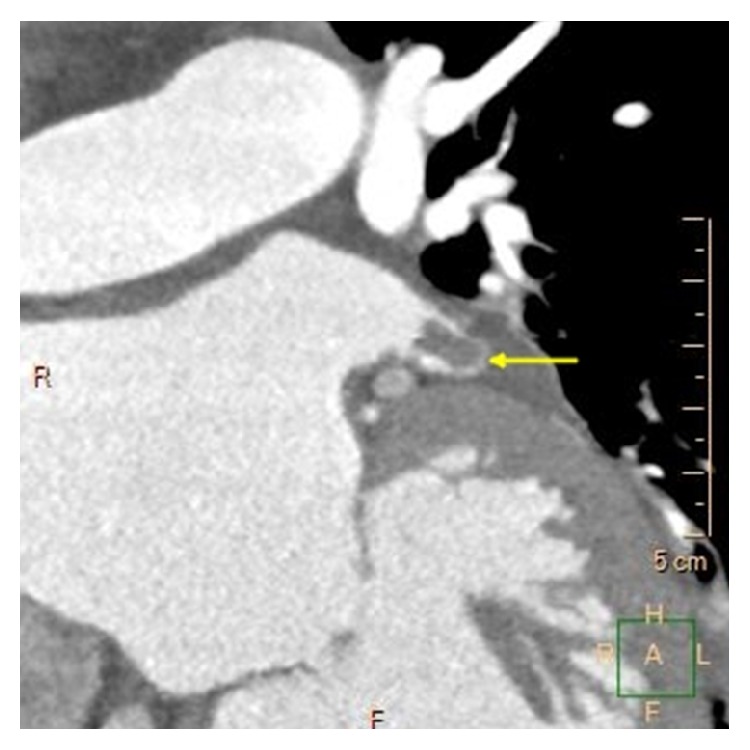
ECG-gated contrast-enhanced CT image depicting a thrombus in the left atrial appendage (arrow).

On CT, a thrombus appears as a homogeneous, low-attenuation lesion that classically does not enhance (Figure 7). However, an old thrombus may be organized, manifesting with a more heterogeneous appearance, with peripheral ring enhancement reflecting the formation of a fibrous capsule, or it may display high-attenuation areas corresponding to internal calcifications.

In various studies, different results in terms of sensitivity and specificity of intracardiac thrombus detection have been reported. Wu et al. performed a meta-analysis to explore the potential diagnostic value of CT in detecting LA or LAA thrombosis [78]. A total of 9 studies with 1646 patients were included in this meta-analysis. The mean CT sensitivity for identifying thrombus in the LA was 81% and the mean specificity 90%. The authors of this meta-analysis concluded that CT should be considered the best noninvasive alternative to TOE for detection of LA/LAA thrombosis.

The main issue in CT is to differentiate between true thrombi and a filling defect corresponding to circulatory stasis. Feuchtner et al. suggested four characteristic imaging features on cardiac CT that could be helpful in characterizing an incomplete filling defect of the LAA corresponding to stasis. The first of these features is the flow phenomenon, defined as the inhomogeneous appearance of mixed blood and contrast agent; the second feature is hypostatic layering, which is a sharp horizontal borderline between the contrast agent and nonmixed blood; the third feature is Hounsfield unit (HU) runoff, with decreasing density dorsally to ventrally; and the last feature is higher intralesional density in comparison with the density of thrombi (153.8 ± 71 versus 71 ± 46.6) [79]. In this study, a density threshold of 60.7 HU was able to differentiate a thrombus from an artefact with a diagnostic accuracy of 97%.

In order to improve the diagnostic accuracy of cardiac CT, Hur et al. suggested performing two-phase cardiac CT angiography [80]. Although this approach was associated with significant improvement in sensitivity and specificity (100% and 98%, resp.), it was also accompanied by higher radiation exposure due to the additional delayed enhanced scan. To avoid the higher radiation dose used with two-phase CT angiography while maintaining a high level of diagnostic accuracy, the same authors developed a new dual-enhanced single-phase CT angiography protocol [81]. This method is based on two injections of contrast agent, with a single scan performed in the late phase, 180 seconds after administration of the first contrast bolus. This study included 83 subjects, with an overall sensitivity and specificity of 96% and 100%, respectively, for the detection of thrombi and circulatory stasis in the LAA using CT.

Furthermore, quantitative analysis of the LAA/ascending aorta (AA) HU ratios was performed. The mean HU ratios significantly differed between thrombus and circulatory stasis (0.15 versus 0.27; *P* = 0.001). A cut-off value of 0.2 was most helpful in distinguishing between these two phenomena; a filling defect with a HU ratio below 0.2 is likely to be a thrombus. Two years later, the utility of quantitative analysis of the LAA/AA HU ratio was confirmed in a study of 101 consecutive patients with atrial fibrillation [82]. Moreover, this study showed that LAA/AA HU ratios were able to differentiate between grades of spontaneous echo contrast detected by TOE.

### 3.5. Left Atrium and Pulmonary Veins

With its ability to acquire a three-dimensional dataset accurately depicting LA and pulmonary venous anatomy, cardiac CT is currently the most commonly used imaging modality for pulmonary vein assessment in many centres, especially if interventional AF treatment is planned.

**Figure 8:**
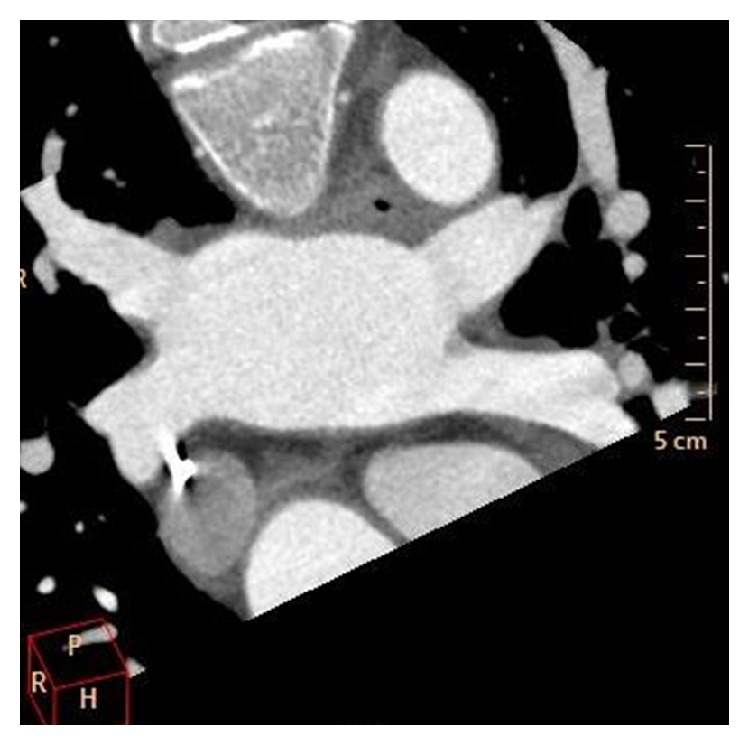
Contrast-enhanced CT scan showing two left-sided and two right-sided pulmonary veins.

Approximately 70% of the general population has the classical configuration of four pulmonary veins: left superior and inferior pulmonary veins and right superior and inferior pulmonary veins, with four independent ostia (Figure 8) [83]. Pulmonary venous variations or abnormalities are related to the complex development of the venous system during first weeks of gestation. The most frequent abnormalities are left-sided pulmonary veins with a common ostium and a third right-sided pulmonary vein [84]. Pulmonary vein ostia have variable diameters, usually ranging between 9 and 13 mm in patients without AF [85] and between 12 and 24 mm in individuals with AF undergoing AF ablation procedure [86].

The clinical utility of CT imaging prior to AF ablation procedures includes preablation imaging of the LA and pulmonary veins to exclude intra-atrial thrombosis and to detect anatomical variants; depiction of the anatomical relationship between the LA, oesophagus, and adjacent vascular structures; creation of a 3D dataset of the LA and pulmonary veins for fusion with electroanatomical mapping data; assessment of morphological remodelling of the LA and pulmonary veins; and, finally, acquisition of a dataset useful for the detection of postprocedure complications (e.g., pulmonary vein stenosis) [35].

The presentation and clinical course of pulmonary vein stenosis vary widely from asymptomatic to highly symptomatic cases. Symptoms usually correlate with the number of the stenotic veins, the severity of the stenosis, the time course of the stenosis, compensatory mechanisms, and development of collaterals. The frequency of right superior, left superior, and left inferior pulmonary veins involvement is similar. The right inferior and right middle pulmonary veins are infrequently involved [87]. Minor stenoses that are defined as <50% luminal narrowing usually show no further progression and are associated with favorable outcome [88]. For detection and grading of the pulmonary vein stenosis severity after AF ablation procedure, the knowledge of the initial pulmonary vein diameters seems to be essential.

### 3.6. Left Atrial Tumours

Cardiac CT represents an alternative or complementary technique to echocardiography and CMR for the characterization of masses in the LA.

**Figure 9:**
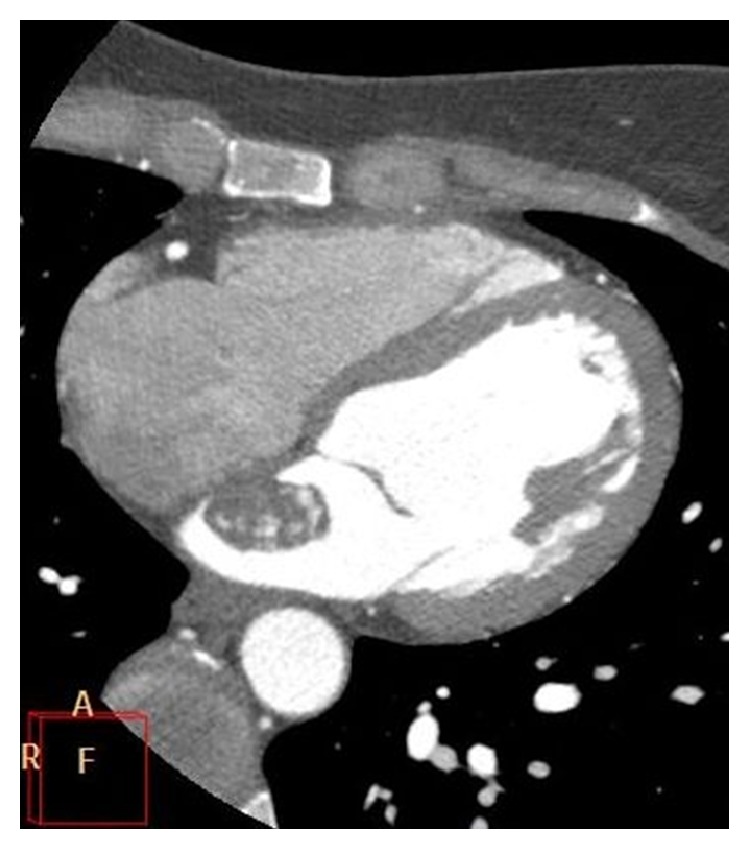
Contrast-enhanced CT image depicting left atrial myxoma attached to the interatrial septum.

Myxomas are the most common primary cardiac tumours, predominantly localized in the LA. Typically, myxomas are solitary, pedunculated lesions attached to the interatrial septum (Figure 9). In unenhanced scans, myxomas have lower attenuation compared with surrounding blood, and their attenuation is usually heterogeneous. On contrast-enhanced scans, this tumour is usually a heterogeneously enhancing structure, with varying degrees of attenuation corresponding to areas of hemorrhage, necrosis, cyst formation, and calcification [77].

Secondary cardiac tumours are at least thirty times more common than primary tumours. Metastases to the heart are mostly associated with lung and breast tumours. Heart involvement is also frequently associated with the progression of various types of lymphomas. Features suggestive of a malignant mass in the LA are a filling defect with poorly defined margins, a lesion crossing tissue planes, an associated pericardial effusion, or the presence of an extracardiac tumour. Metastases are usually multiple and in most cases they involve pericardium and, less frequently, the myocardium or endocardium. Isolated LA involvement is uncommon in secondary tumours.

## 4. Conclusions

Both CMR and cardiac CT currently represent very important imaging modalities used for the comprehensive evaluation of the LA in various clinical settings. Cardiac magnetic resonance is considered to be the gold standard for volumetric assessment of left atrial size. Late gadolinium enhancement CMR offers unique information about the presence of LA scaring that may be used for optimizing the treatment of patients suffering from atrial arrhythmias. Cardiac CT is regarded as the method of choice for evaluation of pulmonary vein and LA anatomy before catheter ablation procedures for AF and also the most useful imaging modality for detection of pulmonary vein stenosis after RFA. Cardiac CT represents an alternative to TOE in exclusion of LA thrombosis. Cardiac magnetic resonance as well as CT also plays very important role in characterizing LA morphological variants and pathological intra-atrial masses.
